# pH-Induced Local Unfolding of the Phl p 6 Pollen Allergen From cpH-MD

**DOI:** 10.3389/fmolb.2020.603644

**Published:** 2021-01-12

**Authors:** Florian Hofer, Anna S. Kamenik, Monica L. Fernández-Quintero, Johannes Kraml, Klaus R. Liedl

**Affiliations:** Center for Molecular Biosciences Innsbruck, Institute for General, Inorganic and Theoretical Chemistry, University of Innsbruck, Innsbruck, Austria

**Keywords:** pollen allergens, Phl p 6, local unfolding, constant pH MD, proteolytic degradation

## Abstract

Susceptibility to endosomal degradation is a decisive contribution to a protein's immunogenicity. It is assumed that the processing kinetics of structured proteins are inherently linked to their probability of local unfolding. In this study, we quantify the impact of endosomal acidification on the conformational stability of the major timothy grass pollen allergen Phl p 6. We use state of the art sampling approaches in combination with constant pH MD techniques to profile pH-dependent local unfolding events in atomistic detail. Integrating our findings into the current view on type 1 allergic sensitization, we characterize local protein dynamics in the context of proteolytic degradation at neutral and acidic pH for the wild type protein and point mutants with varying proteolytic stability. We analyze extensive simulation data using Markov state models and retrieve highly reliable thermodynamic and kinetic information at varying pH levels. Thereby we capture the impact of endolysosomal acidification on the structure and dynamics of the Phl p 6 mutants. We find that upon protonation at lower pH values, the conformational flexibilities in key areas of the wild type protein, i.e., T-cell epitopes and early proteolytic cleavage sites, increase significantly. A decrease of the pH even leads to local unfolding in otherwise stable secondary structure elements, which is a prerequisite for proteolytic cleavage. This effect is even more pronounced in the destabilized mutant, while no unfolding was observed for the stabilized mutant. In summary, we report detailed structural models which rationalize the experimentally observed cleavage pattern during endosomal acidification.

## Introduction

Immunoglobulin E (IgE)-mediated allergies are one of the most prominent health issues in western countries, with more than 20% of the population being affected (Valenta, 2002; Thalhamer et al., 2010; Valenta et al., 2010; Curin et al., 2018). Over 40% of the patients suffer from plant pollen induced allergy, rendering it the most common allergy type in industrialized countries (Asam et al., 2015). Despite the extensive research efforts which have been undertaken in this field, it is still unclear why some proteins induce an allergic immune response in predisposed individuals. Moreover, it was reported repeatedly that slightest distinctions in sequence and/or structure suffice to shift the immune reaction from an allergic to a protective one (Scheurer et al., 2015; Verhoeckx et al., 2019). Moreover, it is known that high similarity in sequence and/or structure to an allergen protein does not necessitate a similar immune response (Mitropoulou et al., 2018; Eichhorn et al., 2019; Seutter Von Loetzen et al., 2019; Tscheppe et al., 2020).

One step, however, which critically influences the immune response and appears to be shared by all protein antigens, is their proteolytic digestion by endolysosomal proteases. The susceptibility of the antigen to degradation, i.e., its proteolytic stability, is linked to the subsequent immune response (Toda et al., 2011; Apostolovic et al., 2016; Machado et al., 2016; Scheiblhofer et al., 2017; Wolf et al., 2018).

After uptake (in case of plant pollen mostly via inhalation) the antigen enters an antigen-presenting cell. In the endosome compartment, proteases digest the antigen into peptide fragments over the course of the endosome maturation. These fragments are then loaded onto class II major histocompatibility complex receptors (MHCII) and are subsequently presented on the cell surface to naïve T-cells (Freier et al., 2015; Machado et al., 2016; Scheiblhofer et al., 2017).

The maturation of the endosome is accompanied by a sharp decrease in pH from approximately 7–4. This decrease in pH can destabilize the protein fold of the antigen and facilitates digestion. It has been previously described that the more stable the fold of the antigen, the lower the pH needs to be so that the antigen unfolds and is processed (Egger et al., 2011; Compeer et al., 2012; Freier et al., 2015; Scheiblhofer et al., 2017). It was suggested that the immune response varies depending on the stability of the antigen. Very unstable proteins are digested already in the early endosome, whereas very stable antigens cannot be processed until a very low pH is reached. The T-cell polarization leading to allergic sensitization, however, can only be achieved if the degradation occurs at a certain maturation state of the endosome, which is associated with moderate pH levels (Scheiblhofer et al., 2017).

This hypothesis was fostered in a comprehensive study by Machado et al. (2016). In this study various point mutations were introduced to the major birch pollen allergen Bet v 1. These mutations were found to alter the thermal and proteolytic stability of the proteins and thereby directly influenced the resulting immune response. Similar effects were also observed for different isoforms of the Bet v 1 allergen, which are characterized by quasi-identical structures as well as very high sequence identities but very different immune responses (Grutsch et al., 2017). Kamenik et al. extended these findings with atomistic models of the unfolding process (Kamenik et al., 2020). From extensive MD simulation data, they could demonstrate how the subtle changes in the allergen's sequence shift the equilibrium populations toward or away from the unfolded conformational state. They further found that the observed differences in ensemble populations rationalize the distinct proteolytic susceptibility of each Bet v 1 variant.

Even more recently, Weiss and coworkers showed consistent results for the major timothy grass pollen allergen Phl p 6 (Winter et al., 2020). Phl p 6 is one of the most important grass pollen allergens, with more than 75% of patients allergic to grass pollen, being allergic to Phl p 6 (Vrtala et al., 1999, 2007). Various point mutations were identified, which lead to differences in thermal and proteolytic stabilities of Phl p 6 (Winter et al., 2020). The proteolytic digestion of thermally stabilized proteins was much slower compared to the wild type and vice versa, while the degradation patterns and resulting peptides remained constant. Furthermore, the stability differences were also found to propagate into the resulting immune response. The increase in stability was further linked to a rigidification of the fold dynamics (and vice versa) with the use of molecular dynamics (MD) simulations (Winter et al., 2020). While already observed encouraging trends with the applied MD-based approach, we neglected the impact of endolysosomal acidification in this previous study. However, as the pH decreases, the protonation state preferences within the protein shift, which can induce massive changes in the electrostatic interaction network within the protein (White and Anfinsen, 1959; Tanford, 1961; Perutz, 1978; Garcia-Moreno, 2009; Di Russo et al., 2012). Hence, it is reasonable to assume that acidification substantially impacts the allergen's conformational ensemble and thus fold stability.

Consequently, in order to profile the influence of lower pH values on the fold stability, it is imperative to perform simulations at lower pH value, i.e., using a protonation state ensemble corresponding to lower pH values. As with classical force fields bonds can neither be broken nor formed, direct sampling of protonation/deprotonation events is not possible. Instead, fixed protonation states are employed, which must be chosen at setup and cannot be changed during the simulation (Chen et al., 2014). Various prediction tools are available to do this, however when moving away from pH 7.0 this becomes challenging very quickly, as sidechain pKa values of amino acids can be strongly shifted depending on the surrounding electrostatic environment within the protein (Harris and Turner, 2002; Alexov et al., 2011; Platzer et al., 2014; Hofer et al., 2019). Capturing these effects can be difficult based on a single, static structure alone (Alexov et al., 2011). But even if all pKa values of the system would be known, an approach with fixed protonation states quickly becomes unfeasible, as the number of titratable residues rises quickly when going to lower pH values and all possible protonation combinations would need to be sampled separately (Chen et al., 2014). Furthermore, the use of fixed protonation states prohibits the sampling of how the titratable residues react to conformational changes and vice versa. The simulations in the aforementioned previous works were indeed performed using fixed protonation states, representing pH 7 and 5 using the respective most probable configuration (Machado et al., 2016; Kamenik et al., 2020; Winter et al., 2020). However, as capturing the effect of the pH on the fold dynamics was not the main focus of these studies, a more detailed treatment of the protonation states was not warranted.

Recently, we employed the so-called constant pH MD (cpH-MD) approach together with NMR titration experiments to profile the protonation states and pKa values of the Phl p 6 wild type (Hofer et al., 2019). These techniques make it possible to sample the conformational as well as the protonation state space at the same time (Chen et al., 2014). Consequently, a structural ensemble along with a proper protonation state distribution is sampled. A more detailed description of these techniques is given in the “Method” section. By furthermore combining the cpH-MD approach with accelerated MD [aMD, (Hamelberg et al., 2004; Williams et al., 2010)] we significantly sped up conformational–and in turn also protonation state–sampling and achieved convergence in all titrated pH values within 1 μs of simulation time. We showed that the combined cpH-aMD approach is stable for long sampling times, while still maintaining a good correlation with experimental reference (Hofer et al., 2019).

Based on these findings, we here use this approach to efficiently sample the conformational space at different pH values of the Phl p 6 wild type as well as two selected point mutations from the dataset presented by Weiss and coworkers mentioned above (Winter et al., 2020). Compared to the wild type (melting point 59.5°C), the S46Y mutant is thermally stabilized (melting point 73.5°C), while the L89G mutant is thermally destabilized (melting point 54.5°C) (Winter_2020). The stabilizing/destabilizing effects of the mutations were also visible in the degradome assay, i.e., the stabilized S46Y mutant remained intact at acidic conditions much longer than the wild type or the L89G mutant. In detail, the wild type and the L89G mutant were only stable at pH 7.0 and showed significant and fast degradation at pH 5.2 and 4.5, the S46Y mutant was only degraded at pH 4.5. Moreover, the cleavage areas reported in the assays as well as the early cleavage peptides were mostly similar for all systems. As both point mutations did not alter the early cleavage positions or their direct surroundings, Weiss and coworkers surmised, that the point mutations indeed influenced the stability of the protein fold or parts of it in general. This was supported by the molecular flexibilities captured with the use of classical MD simulations, which depicted an overall structural rigidification for the S46Y mutant, as well as an overall increase in molecular flexibility for the L89G mutant. The simulations were performed with a protonation ensemble corresponding to pH 7.

In contrast to the workflow used by Kamenik et al., we here use the cpH-aMD approach as described above instead of Metadynamics (Laio and Gervasio, 2008). On the one hand, this removes the need to define a specific collective variable (CV) along which the simulation is accelerated, which in turn does not limit our findings to one specific unfolding pathway. On the other hand, we incorporate the impact of the acidic pH on the dynamics of the systems and are furthermore able to sample the interplay of conformational and protonation changes. Taken together, the focus of the present study is to characterize fold stability and potential local unfolding during endolysosomal acidification without any a priori assumption on the most likely unfolding or cleavage site.

## Methods

### Structure Preparation

**Figure 1:**
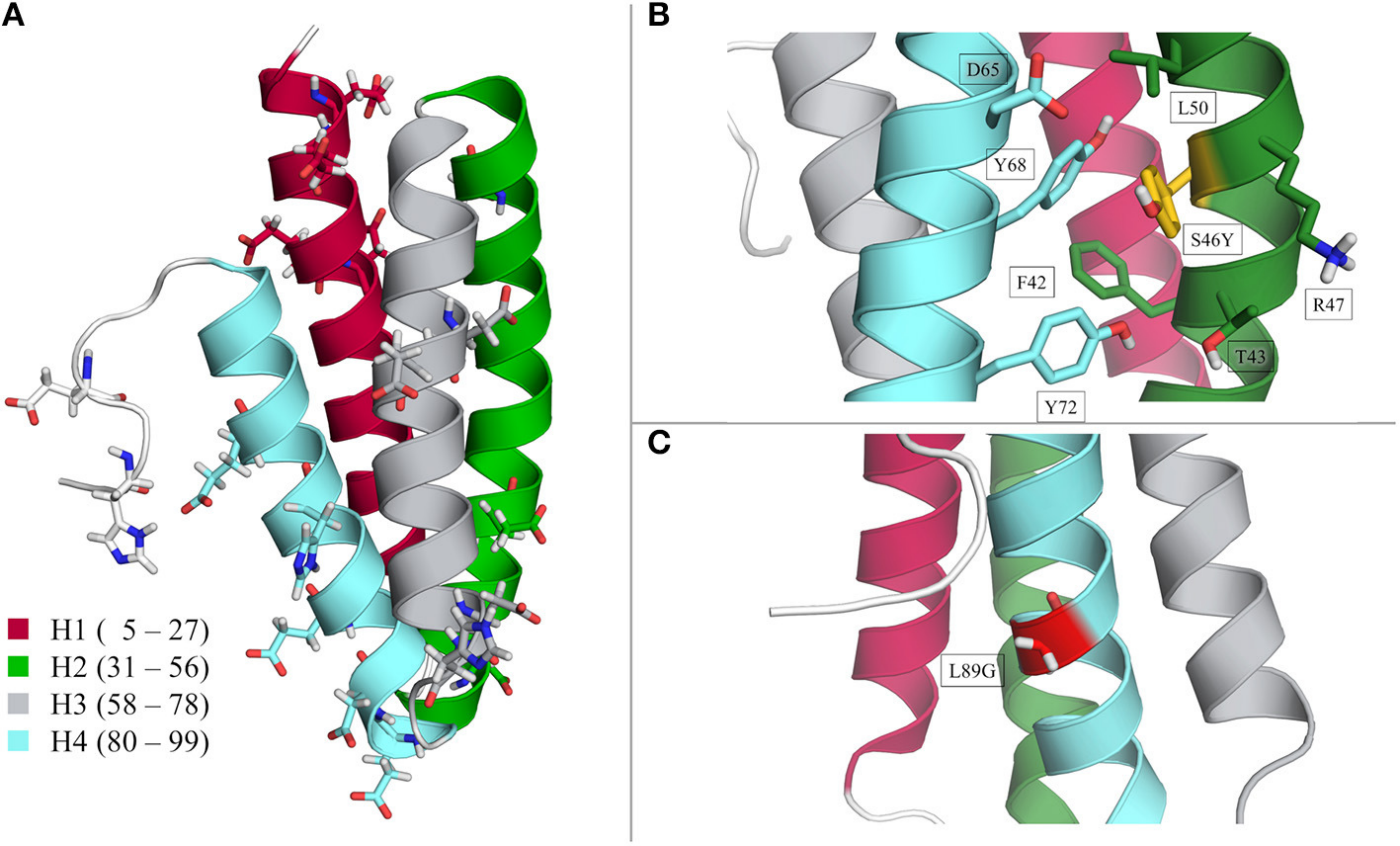
Structural visualization of the Phl p 6 allergen. Wild type crystal structure (PDB 1NLX, chain A) with color-coded helices 1–4 and all titrated residues is in section **(A)**. Location of the S46Y and L89G mutation are shown in sections **(B)** and **(C)**, respectively.

Starting structures for the Phl p 6 wild type as well as for the point mutants S46Y and L89G were prepared from the wild type X-ray structure (PDB Code 1NLX, chain A; 104 of 111 residues resolved) with the program MOE [molecular operating environment (CCG, 2017)]. Point mutations were also introduced with MOE, followed by a short local minimization. The molecular structure is visualized in Figure 1. The protein is composed of four helices, which are connected via short loops. In Figure 1A the wild type structure is shown with color-coded helices for later reference. Furthermore, all the residues which were titrated are visualized in Figure 1A. The locations and possible interaction sites of the S46Y and L89G mutants are shown in Figures 1B,C, respectively. The titrated residues were also visualized in the close-ups of the locations of the point mutations (see Supplementary Figure 9).

With the LEaP program of AmberTools 19 (Case et al., 2019) missing hydrogens were added and topology and starting coordinates were created. The AMBER ff99SB force field (Lindorff-Larsen et al., 2010) with the modifications needed for the cpH-MD approach were used (Mongan et al., 2004; Swails and Roitberg, 2012; Swails et al., 2014). As suggested by Swails et al., the GB radii of the titratable oxygens of Aspartate and Glutamate were changed to 1.3 Å (Swails et al., 2014). All systems were placed in a truncated octahedral TIP3P water box (Jorgensen et al., 1983), with a 10 Å padding. Before production simulation, all systems were equilibrated with an elaborate protocol (Wallnoefer et al., 2010).

### Simulation Setup and Theory

#### Constant pH MD Simulations

Generally, there are two different approaches to constant pH MD, differing in the treatment of the protonation states, i.e., the titratable protons. On the one hand continuous protonation states can be used, which are sampled along a continuous titration coordinate λ (Lee et al., 2004; Huang et al., 2018). When using discrete protonation states on the other hand, the simulation is interrupted at periodic intervals and protonation changes are attempted based on a Monte Carlo Metropolis criterion (Baptista et al., 2002; Mongan et al., 2004; Stern, 2007; Swails and Roitberg, 2012). In this study, we used the latter concept, specifically the most recent implementation for explicit solvent in the AMBER package by Roitberg and coworkers (Swails et al., 2014). In the following a short outline of the approach is given, for a more detailed description of the methods and their respective limitations the reader is pointed to the respective publications (Baptista et al., 2002; Lee et al., 2004; Mongan et al., 2004; Stern, 2007; Swails and Roitberg, 2012; Swails et al., 2014; Radak et al., 2017; Huang et al., 2018).

In brief, at each titratable group, the titratable protons are defined explicitly. A state list is defined for each residue, detailing which proton is active or inactive in which state. The simulation itself runs with fixed protonation states but is interrupted periodically and protonation state changes are attempted based on a Metropolis criterion. If at least one state change is accepted, the protein is frozen and the solvent around it is relaxed for a predefined number of steps. Hereafter, the simulation continues with the new protonation states until the next change is attempted (Mongan et al., 2004; Swails et al., 2014).

#### Accelerated MD Simulations

Accelerated MD simulations are an enhanced sampling technique, which modify the underlying potential energy surface by adding a so-called boost potential to the system, should the system's energy be under a certain threshold (Hamelberg et al., 2004). There exist various implementations, in this paper we employed the so-termed double boost mode as implemented in AMBER 18 (Case et al., 2019). Here, not only the total energy is boosted, but the dihedral energy receives an additional boost energy. Again, we point the reader to the original works for a more in-depth discussion of the method (Hamelberg et al., 2004). The exact boosting parameters and how they were obtained is described in the supporting information.

Combining aMD with the cpH-MD framework, allows for faster conformational sampling, which in turn also speeds up the protonation state sampling, as this also depends on the sampled conformations. Moreover, the sampling of the response of the system to a lower pH value is accelerated and can be captured within reasonable sampling times (Williams et al., 2010; Hofer et al., 2019).

#### Seeding of Classical Simulations

As mentioned above, the bias potential introduced in the aMD approach modifies the underlying potential energy surface. To obtain accurate thermodynamic and kinetic data the original free energy profile must be reconstructed, by reweighting the obtained trajectories (Sinko et al., 2013; Miao et al., 2014; Stelzl et al., 2018). While this is generally possible and straightforward, most of the proposed reweighting techniques inherently lead to very noisy energy profiles. We circumvent this problem by seeding classical MD simulations from the captured ensembles. We performed a hierarchical clustering of each ensemble using the backbone heavy atom cartesian coordinates as input and a cutoff of 2.0 Å. We obtained a varying number of clusters for each system and each pH value and started a 200 ns long classical cpH-MD simulation from each cluster representative. We then combined the resulting trajectories for each pH and each system respectively with the use of Markov State Models (MSMs) (Prinz et al., 2011). This approach removes any bias stemming from a different number of starting clusters in the seeding process and allows us to capture accurate thermodynamic and kinetic properties of the sampled dynamic processes (Bowman et al., 2013; Kohlhoff et al., 2014). Similar approaches have been reported previously (Noé et al., 2009; Nedialkova et al., 2014; Biswas et al., 2018; Sun et al., 2018; Zimmerman et al., 2018; Fernández-Quintero et al., 2019a,b, 2020; Kahler et al., 2020; Kamenik et al., 2020).

#### Simulation Parameters

All simulations were performed with the GPU implementation of the pmemd module of AMBER 18 (Case et al., 2019). A Langevin thermostat with a collision frequency of 5 ps^−1^ was used to keep a constant temperature of 310 K (Adelman and Doll, 1976). Constant pressure of 1 bar was maintained with a Berendsen barostat with a pressure relaxation time of 2 ps (Berendsen et al., 1984). Long range electrostatics were treated with the Particle-mesh Ewald approach and a non-bonded cutoff of 8 Å was used (Darden et al., 1993). To allow for a time step of 2 fs, all bonds involving hydrogen were restrained with the SHAKE algorithm (Ryckaert et al., 1977). Frames were collected every 2 ps.

Constant pH aMD simulations were run at pHs 4.0–7.0 with a 1.0 spacing. Protonation state changes were attempted every 200 steps, succeeded by 200 steps of solvent relaxation, should at least one attempt be successful. A salt concentration of 0.1 M was used (Swails et al., 2014). Acceleration was achieved with the dual boost option as available in AMBER 18 (Case et al., 2019). Acceleration parameters were derived as suggested by Pierce et al. (2012). Each simulation was run for 1 μs.

Seeded cpH simulations were started from the cluster representatives as described above, with the same parameters as the cpH-aMD simulations, except for the acceleration. Each simulation was run for 200 ns.

### Analysis

Trajectories were processed and analyzed using cpptraj and pytraj from the AmberTools 19 package (Case et al., 2019) and in-house python scripts. PCA, TICA, and MSM analysis was done with the PyEMMA package version 2.5.7 (Scherer et al., 2015). Structural visualizations were done with PyMol (Schrodinger, 2019).

While most of the Phl p 6 structure is helical, the C-terminal part (residues 101 onwards) is an unstructured and consequently very flexible loop. As the strong dynamics of this loop would dominate all analyses, we excluded this part from all analyses, unless otherwise noted. Furthermore, all alignment operations were based on residues 1–98 to focus on the dynamics of the core of the protein.

Structural variances were primarily analyzed and visualized with the use of principal component analysis (PCA) based on the heavy atom backbone cartesian coordinates, supplemented by RMSD, DRMSD, secondary structure, and native contacts analyses. A combined PCA space was constructed by combining the trajectories of all simulated systems at all pH values. Projecting the individual trajectories into the combined space allowed for a direct characterization of the impact of the pH on the dynamics of the system.

Time-lagged independent component analysis (TICA) was used to identify the slowest movements in the systems and thus obtain a kinetic discretization of the sampled spaces (Molgedey and Schuster, 1994; Chodera and Noé, 2014; Schwantes et al., 2016). For the TICA analyses, the same inputs were used as for the PCAs, with a lagtime of 100 ns for each system. Based on the TICA spaces, Bayesian Markov State Models (MSMs) were constructed to characterize the thermodynamics and kinetics of the captured local unfolding and refolding events (Bowman et al., 2013; Shukla et al., 2015). For all constructed models a lagtime of 100 ns was used. The constructed models were further coarse grained with the use of Perron cluster cluster analysis (PCCA+) (Röblitz and Weber, 2013). The number of used clusters depended on the system and the analyzed pH value. Reliability of the models was validated by implied timescale analyses and Chapman–Kolmogorov tests.

## Results

We use a combination of double boost aMD and constant pH simulations to profile local unfolding events of the Phl p 6 WT and two point-mutants at pH values ranging from 4.0 to 7.0.

**Figure 2:**
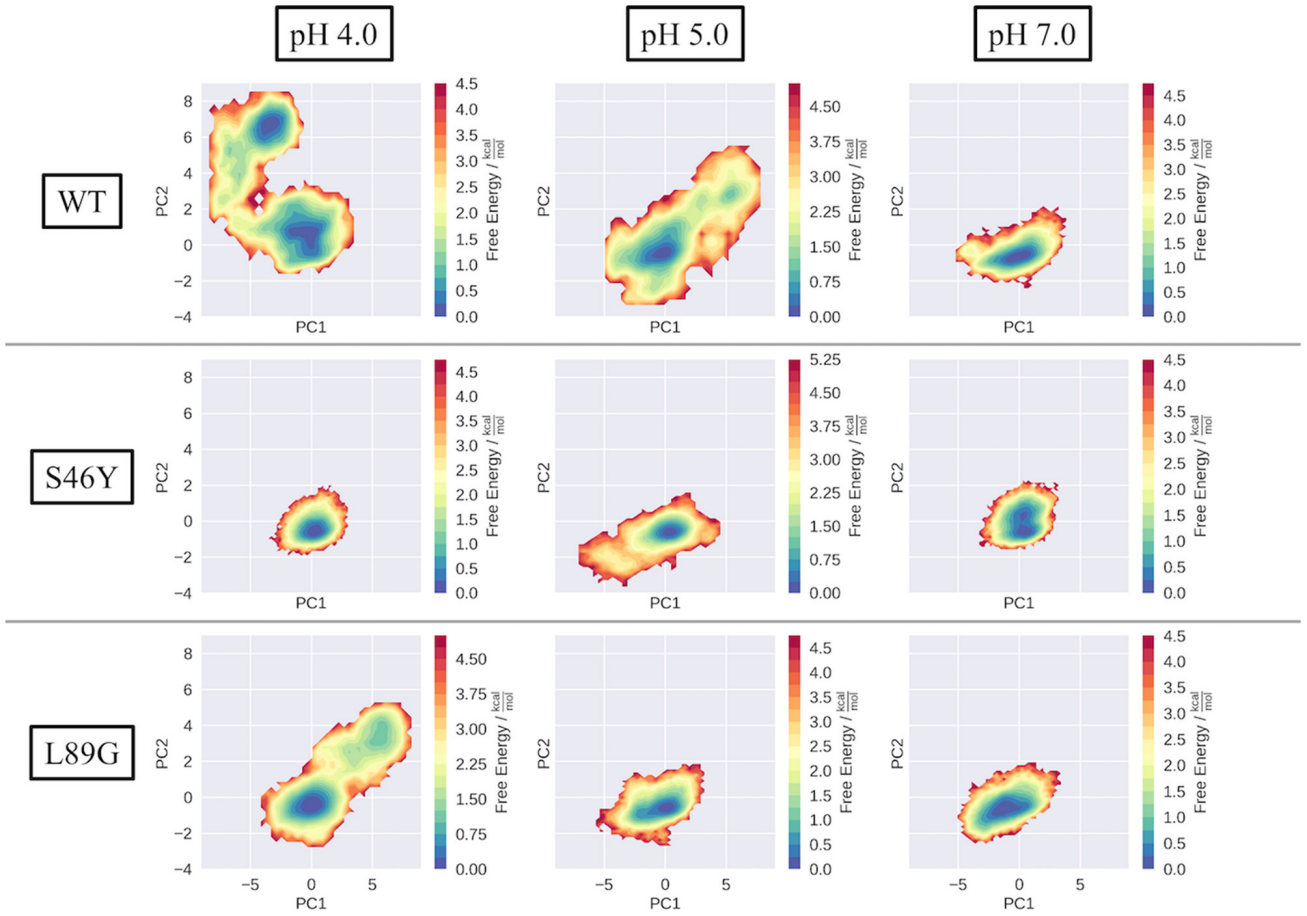
Captured conformational space of the WT, S46Y, and L89G mutant. Combined PCA space was constructed from all simulations. Individual simulations are projected onto the first two principal components and color-coded according to the reweighted free energy.

To compare the structural variations found in the simulations, we projected each simulation into a PCA space, constructed from the combined trajectories of all systems at all pH values (Figure 2 WT). The three systems show strong differences in the captured dynamics. While the WT is stable at pH 7.0, with a single, deep minimum in the PCA corresponding to the folded state, the simulations get more and more flexible as the pH value decreases. The WT simulation of pH 4.0 shows the broadest surface across all systems, with two distinct minima in free energy. One of the minima corresponds to the folded structure, very similar to the simulation at pH 7.0. The second distinct minimum represents a sub-ensemble of unfolded structures, which are comparably high populated at this pH value. Secondary structure and DRMSD analyses paint a similar picture, i.e., the simulation at pH 7.0 is stable in all metrics, showing only little fluctuations and unfolding, while at lower pH values the flexibility and structural variances increase significantly (see Supplementary Figures 1, 2).

In contrast to the WT, the stabilized S46Y mutant, exhibits limited flexibility in all simulations. Even at lower pH values, the overall structure remains stable and shows no indications of unfolding, as represented by the single deep minimum in the PCA space (Figure 2, S46Y). Only at pH 5.0, the captured space is broader, however the sampled states are comparably high in free energy. The 2D-RMSD plot of this simulation shows a notable, yet short-lived conformational change at about 300 ns of simulation time. However, the structure quickly refolds and remains stable for the rest of the simulation.

The destabilized L89G mutant, exhibits a similar behavior as the WT in the simulations (Figure 2, L89G). Again, the sampled space clearly gets broader at lower pH values, however even at pH 4.0 the unfolding does not appear to be as strong as in the WT simulations, as no deep second minimum appears in the PCA space. Nevertheless, clear unfolding tendencies are visible at pH 4.0, with a rather shallow free energy profile (apart from the dominant minimum) and comparably low barriers.

In order to obtain accurate thermodynamic and kinetic information of the unfolding processes captured in our simulations, we clustered the trajectories of pH 4.0 and 7.0 and used the cluster representatives as starting structures for classical cpH-MD simulations (see “Method” section). The number of obtained seeds for the various systems is shown in Table 1. The varying number of obtained clusters is consistent with the PCA and flexibility analyses described above, showing little diversity in the S46Y ensemble on both pH values and an overall higher and more pH dependent diversity for the WT and the L89G mutant. For each system and pH, the resulting trajectories were projected into the original PCA space constructed from the combined cpH-aMD trajectories and further analyzed with Markov State Models. The use of MSMs removes the bias originating from the different number of starting structures and allows for a reliable estimation of the thermodynamics and timescales of the captured unfolding processes. A summary of all state probabilities and all estimated transition timescales is available in the supporting information (Supplementary Tables 1–7).

**Table 1:** Number of obtained clusters from the 1 μs long cpH-aMD trajectories, for all systems, at all simulated pH values.

**System**	**pH 4.0**	**pH 4.5**	**pH 5.0**	**pH 7.0**
WT	348	114	219	54
S46Y	37	31	67	33
L89G	172	161	55	92

**Figure 3:**
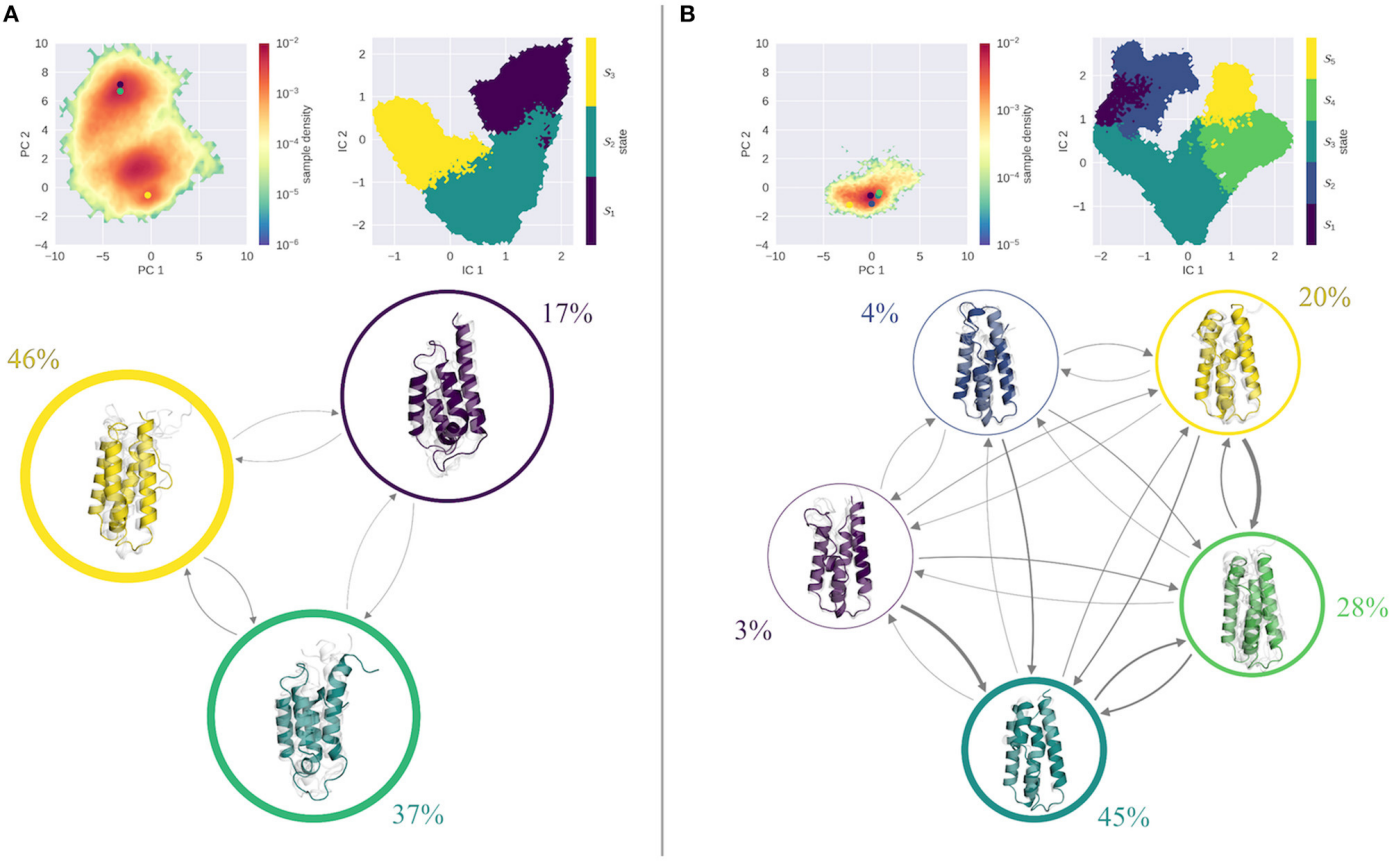
MSM analysis of the seeded simulations of the WT at pH 4.0 **(A)** and pH 7.0 **(B)**. Simulations were projected into the combined PCA space, showing the reweighted free energy surface, together with the positions of the representative macrostate structures. State separation is shown in the respective TICA space. Macrostate and transitions are visualized, where width of the surrounding circle represents state population and arrow thickness denotes the transition timescale. Colored structure denotes ensemble representative of the specific macrostate, while transparent, gray structures illustrate ensemble members. At pH 7.0 primarily folded states are populated, with fast transitions into them. At pH 4.0, folded and unfolded states are populated equally with comparable transition timescales in between.

Figure 3 shows the results of the MSM analysis of the WT at pH 4.0 (Figure 3A) and pH 7.0 (Figure 3B). To visualize the captured dynamics, we projected the sampled space into the original PCA space of the combined cpH-aMD trajectories, and color-coded it according to the MSM-reweighted free energy. We also mapped the representative structures of the macrostates into the same space. Furthermore, the macrostate separation in the TICA space is shown. The seeding process allowed for a broad sampling of the transition regions between the two main minima at pH 4.0 as shown in Figure 3A. Macrostates 1 and 2 can be attributed to the upper minimum [located around (−3,7) in the PCA space), while macrostate 3 corresponds to the lower minimum [around (0,0)]. Macrostate 3 reveals the highest stationary probability of 46%, macrostates 1 and 2 are less probable, with a stationary probability of 17 and 37%, respectively. The estimated transition timescales are in line with these observations, as the back-and-forth transitions between states 2 and 3 are comparably fast (~60 μs), while transitions to state 1 are comparably slow (~1 ms). Structurally, the three macrostates are characterized by a varying degree of unfolding. While the structural ensemble of state 3 is primarily folded, states 1 and 2 clearly contain partially unfolded structures with varying severity.

The simulations at pH 7.0 show a shallow minimum in the combined PCA space, very similar to the cpH-aMD simulations (see Figure 2). The MSM analysis yields 5 macrostates, three of which are estimated to contain over 90% of the analyzed frames (states 3, 4, and 5) and two, significantly less populated states. Transitions between the highly populated states are estimated to be quite fast (almost all below 50 μs), whereas transitions to states 1 and 2 are estimated to be in the range of 500−600 μs. Transitions away from the unfavorable states on the other hand are estimated to be much faster, in the range of around 100 μs and below. Structurally, the most populated state 3 shows an overall stable fold in the ensemble representatives, albeit a disturbance of the second helix is clearly visible. In fact, a similar disturbance is visible in all states with varying intensity. For the rest of the protein, only minimal unfolding is visible, with the least populated macrostates 1 and 2 showing the most notable structural distortions.

**Figure 4:**
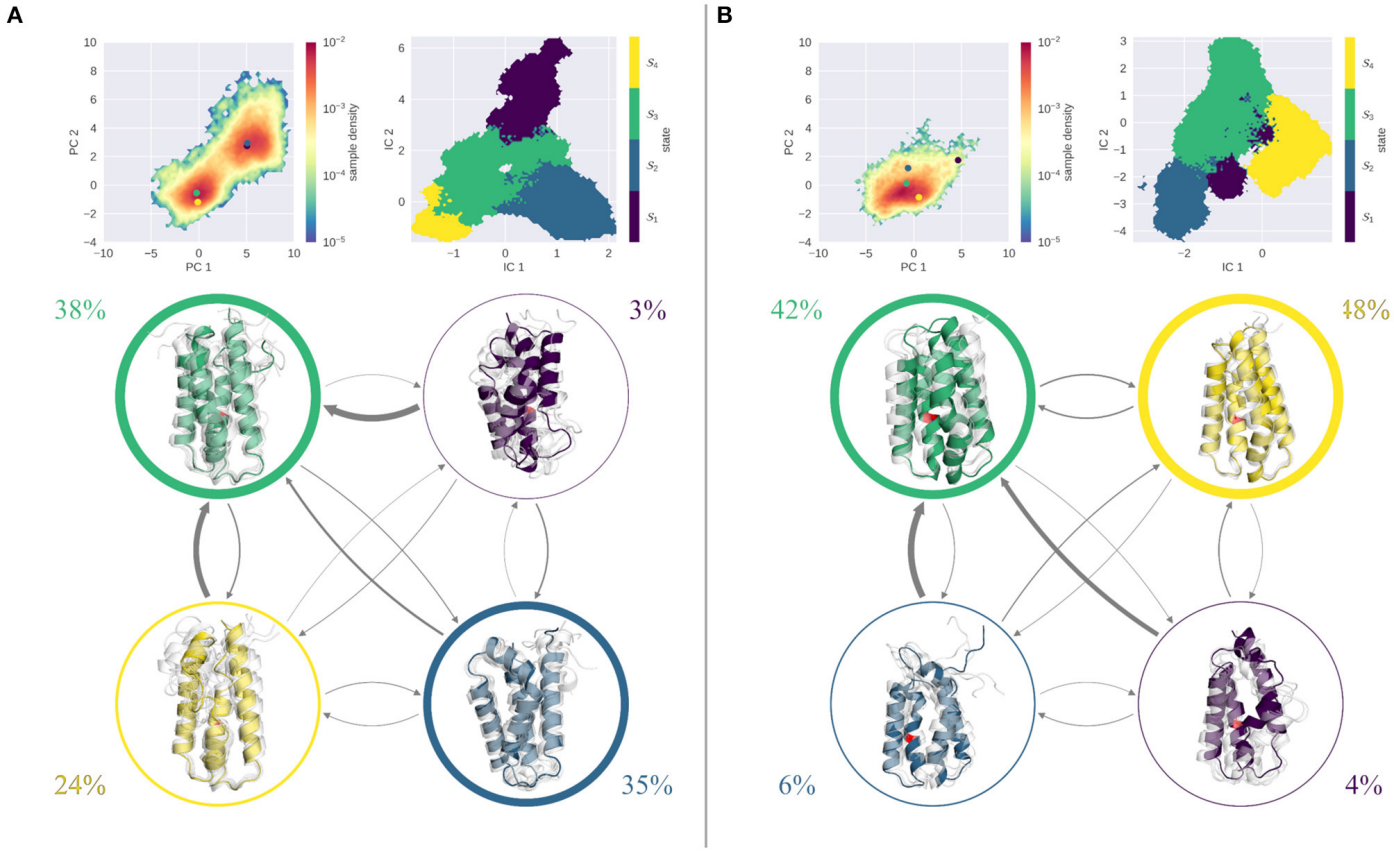
MSM analysis of the seeded simulations of the L89G at pH 4.0 **(A)** and pH 7.0 **(B)**. Simulations were projected into the combined PCA space, showing the reweighted free energy surface, together with the positions of the representative macrostate structures. State separation is shown in the respective TICA space. Macrostate and transitions are visualized, where width of the surrounding circle represents state population and arrow thickness denotes the transition timescale. Colored structure denotes ensemble representative of the specific macrostate, while transparent, gray structures illustrate ensemble members. Already at pH 7.0 partially unfolded states are sampled with reasonably fast transitions in between. At pH 4.0 all macrostates show partial unfolding, most transitions are below 100 μs.

The results of the MSM analysis of the L89G mutant are shown in Figure 4. Again, the sampled space was projected in the combined PCA space and color-coded according to the MSM reweighted free energies. At pH 4.0, two deep minima are visible in the reweighted PCA space. TICA analysis, followed by the construction of an MSM discretizes the TICA space into four macrostates (Figure 4A, right). Mapping these macrostates back into the PCA space, we see that macrostates 1 and 2 are attributed to the minimum around (5,3), while macrostates 3 and 4 correspond to the minimum at (0,0). Clustering of the corresponding structures reveals, that all macrostates exhibit varying degrees of unfolding, with state 1 showing the most fold distortions of the whole ensemble. While this state also shows the lowest stationary distribution, also states 2 and 3, which together hold almost 80% of the population, show clear signs of unfolding. Transitions between states 2–4 are comparably fast, with transition timescales below 100 μs. Transitions to the highly unfolded state 1 are slower (around 300 μs), transitions out of this state are again fast, i.e., below the 100 μs margin.

At pH 7.0 on the other hand, the PCA is characterized by one single minimum, similar to the wild type simulations (see Figure 4B). Kinetically, again 4 states can be differentiated by the MSM analysis, of which states 3 and 4 contain nearly 90% of the ensemble, while states 1 and 2 are significantly less populated. Structurally, both highly populated states are overall stably folded, with only state 3 showing slight disturbances. In contrast, states 1 and 2 exhibit a significant degree of unfolding in all parts of the protein. Mapping the cluster representatives back into the PCA space, we find that the highly populated states 3 and 4 are located near the center of the minimum, while macrostates 1 and 2 are high energy states. Transitions between all states are reasonably fast, with favored transitions into states 3 and 4.

**Figure 5:**
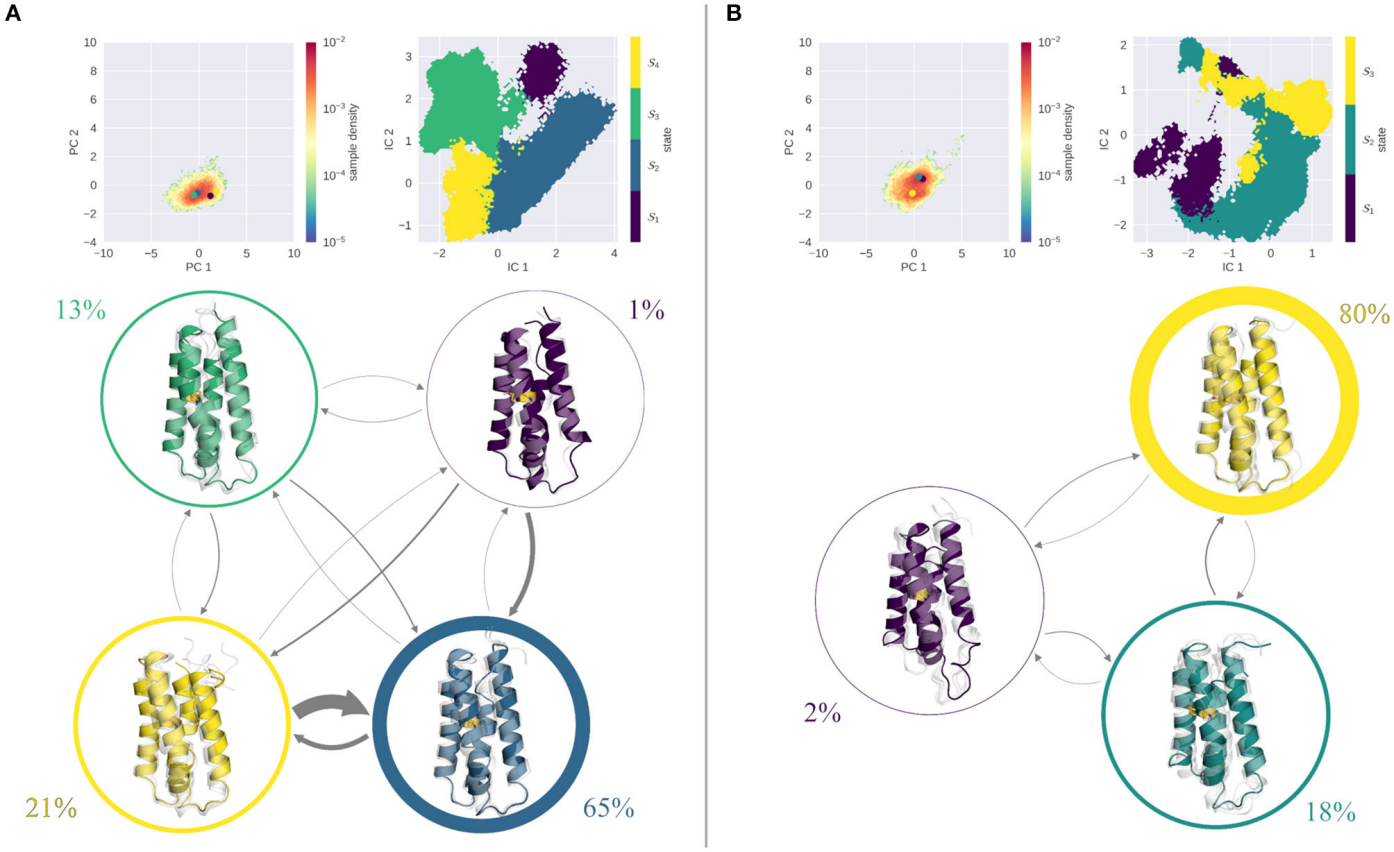
MSM analysis of the seeded simulations of the S46Y at pH 4.0 **(A)** and pH 7.0 **(B)**. Simulations were projected into the combined PCA space, showing the reweighted free energy surface, together with the positions of the representative macrostate structures. State separation is shown in the respective TICA space. Macrostate and transitions are visualized, where width of the surrounding circle represents state population and arrow thickness denotes the transition timescale. Colored structure denotes ensemble representative of the specific macrostate, while transparent, gray structures illustrate ensemble members. Fold remained stable at pH 7.0, the single partially unfolded macrostate is small in population, and transition timescales into it are above 4 ms. At pH 4.0 transitions are faster, however transitions into the folded state (3) are much faster than transitions to an unfolded state.

Figure 5 shows the results of the seeded simulations for the stabilized S46Y mutant. As described above, the S46Y mutant retained a stable fold at all pH values in the cpH-aMD simulations, showing little flexibility and structural changes. This is emphasized by the comparably low number of seeds obtained from the clustering of the cpH-aMD trajectories (see Table 1). Additionally, the seeded simulations did not increase the sampled space significantly, as is evident from projections into the PCA space. At both pH values, only one deep, narrow minimum is sampled. At pH 4.0 (Figure 5A) TICA and MSM analyses revealed four kinetically separated macrostates. With a stationary probability of 65%, macrostate 2 is the most populated of all states, states 3 and 4 are significantly less populated (13 and 21%, respectively) and state 1 is almost negligible, with <1%. Structurally, all macrostates are folded, only state 1 shows some disturbance in helix 2. Transitions to and between states 2 and 4 are generally very fast (ranging from 45 down to 3 μs), transitions to other states are much slower (up to 400 μs). At pH 7.0 (Figure 5B) even less dynamics are captured in the simulations as illustrated by the three macrostates. State 3 shows by far the highest stationary distribution (80%) and completely retains the native fold. State 2, with a stationary distribution of 18%, has some diversity in helix 2, however overall its fold is stable. State 1 is the only state that shows some degree of unfolding, however its probability is very low (~2%) and transitions to it are extremely slow (above 4 ms). Notably, the dynamics captured on pH 4.0 and on pH 7.0 appear to be very similar for the S46Y mutant and little to no unfolding is sampled in the simulations. This stands in contrast to the WT and the L89G simulations, which both are significantly more flexible at lower pH and extensive unfolding processes are sampled.

## Discussion

We performed constant pH aMD simulations to profile pH induced local unfolding events in the Phl p 6 wild type as well as two point mutants. To increase the statistics and obtain accurate thermodynamics and kinetics of the sampled unfolding processes, we seeded classical constant pH simulations from the aMD trajectories and constructed Markov State Models on the obtained seeded trajectories.

Already in the cpH-aMD simulations clear differences between the simulated systems can be observed (see Figure 2). At pH 7.0 all three systems show limited dynamics in the combined PCA space. Helicity analyses indicate that the wild type and the L89G mutant are more flexible and structurally diverse compared to the S46Y mutant (see Supplementary Figure 1 in the supporting information). This is consistent with the experimental melting temperatures, which show a thermal stabilization of 14°C of the S46Y mutant compared to the wild type (Winter et al., 2020). On the other hand, the slight thermal destabilization of −5°C of the L89G mutant compared to the wild type is less pronounced in our simulations, with both systems showing comparable dynamics in our analyses.

At lower pH values, the stabilization effect of the S46Y mutation becomes even more apparent. The mutant retains an overall stable structure, all four helices remain intact and the DRMSD distributions are quite similar across all pH values (see Supplementary Figures 1, 2). In contrast, the L89G mutant shows considerably more flexibility and indications of partial unfolding, which is further reflected in our clustering analyses as discussed below. The direction of unfolding appears to be different in the PCA projection between the wild type and the L89G simulations (see Figure 2). This can be attributed to a slight displacement of helix 1 relative to the rest of the protein, which is captured in the pH 4.0 WT simulation but not in the L89G simulation. The unfolding captured in the L89G simulation is concentrated on the unfolding of helix 2.

While for the S46Y the number of obtained clusters in our analyses is basically constant between pH 4.0 and pH 7.0, both for the wild type as well as the L89G mutant, a strong increase in the number of clusters can be observed going from pH 7.0 to pH 4.0 (see Table 1). Interestingly, nearly double the amount of clusters was obtained for the L89G mutant at pH 7.0, compared to the wild type. This indicates that while appearing to be quite similar in the other analyses, the captured structural ensemble was still more diverse for the L89G mutant. At pH 4.0 we find the exact opposite, which again is in line with our other observations. Given the importance of allergen stability and proteolytic digestion at lower pH levels, we further focused on the differences between pH values 4.0 and 7.0.

The MSMs (Figures 3–5) clearly show that at pH 7.0 the highly populated states are all mainly composed of folded structures, illustrating the overall stability of all systems at this pH value. Local unfolding events do occur; however, their transition timescales are quite slow and the respective stationary distributions are always very small compared to the folded states. Of all systems the L89G mutant shows the fastest transitions to partially unfolded states. This assessment is supported by the calculated DRMSD distributions (see Supplementary Figure 6, lower panel). While the peaks of the distributions are quite similar, the distribution at pH 7.0 is distinctly broader for the L89G mutant.

This picture changes at pH 4.0. Here, the folded states show a decrease in probability, while unfolded states become more probable. Both the wild type and the L89G mutant show comparably fast transitions (below 100 μs) from the folded to an unfolded state, which is comparable in probability. Especially the L89G shows a highly diverse structural ensemble, comprising multiple local unfolding hotspots, with relatively fast transitions between all states. Similar but not equivalent in its properties, is the captured conformational ensemble of the wild type. As is visible from the stationary distributions, folded and unfolded states are populated almost equally, with transitions below 100 μs between the folded and the dominant unfolded state. Compared to the L89G ensemble, however, the unfolding events in the wild type appear to be less fuzzy and more localized to certain parts of the allergen. Taken together, the expected destabilizing effect of the lower pH value is clearly captured and reproduced by our simulations. While still visible, this pH effect is least pronounced in the S46Y ensemble, as all four macrostates at pH 4.0 show only small disturbances in their fold. However, the ensembles are more dynamic than at pH 7.0, as illustrated by the estimated transition timescales. Again, this is nicely captured in our DRMSD analysis (Supplementary Figure 6 upper panel). The DRMSD distributions show two peaks for the wild type and the L89G mutant, while the S46Y mutant remains stable.

Our findings in this study, as discussed above, are well in line with the reported experimental degradation behavior and discussion of its origins made by Weiss and coworkers (Winter et al., 2020).

To further link our findings to the experimental degradation reports and early cleavage patterns, we focused on the resulting degradation peptides and proteolytic cleavage positions as reported by Winter et al. The reported peptide fragments—and in consequence also the cleavage positions—are remarkably similar for all systems and can be grouped into four separated “clusters” depending on their sequence position. The first and fourth cluster are located at the N-terminus and near the C-terminus, respectively. Clusters two and three, on the other hand, are located within the core of the protein. Taking a closer look at cluster 2, which spans from amino-acid position 45 to around 65, and the involved cutting positions, we note that while the cutting at position 64–65 is very clear and frequent, the N-terminal end point of the peptide is much more variable. Interestingly, this cluster is distinctly less represented in the S46Y degradation pattern. The peptides of cluster 3 (65–85) are less fuzzy and the related cutting positions clearer. For all these fragmentations, a prior local unfolding at and around the cleavage position is necessary. Our simulations depict the second helix (residues 31–56) to be one of the most flexible parts of the whole protein. Especially its C-terminal half is very flexible and shows a strong tendency to unfold, which is well captured and visualized in our MSM analysis of the wild type and the L89G mutant simulations at pH 4.0. Native contact and helical content analyses of helix 2 further support this, showing a significant shift toward a lower fraction of native contacts for the L89G mutant, compared to the other systems (see Supplementary Figures 3–6). We surmise that the local unfolding of helix 2 opens up the protein to proteolytic attack and subsequent fragmentation. The high diversity in the unfolding of these parts is in line with the fuzzy N-terminal cleavage of the second peptide cluster of the wild type and the L89G mutant mentioned above.

Remarkably, the stabilized S46Y mutant shows distinctly less flexibility and little to no unfolding in this region (see again Supplementary Figures 4, 6), which is again consistent with its reported cleavage pattern. This difference in flexibility can be attributed to the stabilizing effect of the serine to tyrosine mutation in this region. The introduction of the tyrosine at position 46 stabilizes the core by the introduction of an additional hydrophobic pi-stacking partner, without removing the hydrogen bonding potential.

The L89G mutation, on the other hand, is most likely not directly related to the unfolding tendencies and degradation patterns of helix 2 due to its position and orientation. Experimentally, the destabilizing effect of the L89G mutant, is primarily reflected by its lower melting temperature and faster degradation at higher pH values compared to the wild type. Notably, the frequency of the first peptide cluster and to a lesser degree the frequencies of clusters 2–4 are increased. Weiss and coworkers attributed these observations to a local increase in flexibility in helix 4, caused by the mutation to glycine, which likely propagates into the surrounding parts of the protein. As discussed above, our results of the DRMSD analyses support this argument (Supplementary Figures 5, 6), attributing a higher flexibility to the L89G mutant at pH 7.0 than the other systems. Furthermore, a native contact analysis of helix 1, to which the first peptide cluster can be attributed, shows a distinct shift to a lower fraction of native contacts in the L89G mutant, which is not captured for the other two systems.

The difference in flexibility and degradation behavior between pH 4.0 and 7.0 found for all systems, can be attributed to the changes in protonation states of the titratable residues. As we have shown previously many of the 18 residues, which we consider titratable in the analyzed pH range, are actually titrating around pH 4.0 (Hofer et al., 2019). In Supplementary Table 8 in the supporting information we compare the average protonation of each titratable residue in the systems at both pH values. We see that most acidic residues show a notable fraction of protonation at pH 4.0, while at pH 7.0 all acidic residues are negatively charged. We surmise that this dramatic change in the charge distribution within the system is the main cause of the strong difference in dynamics between low and high pH. However, we do not deem our approach suitable for a detailed investigation of the pH-induced unfolding mechanism itself. To do that, a dedicated, slow titration study could be performed to slowly adapt the system to the change in pH and monitor its reaction to that.

**Figure 6:**
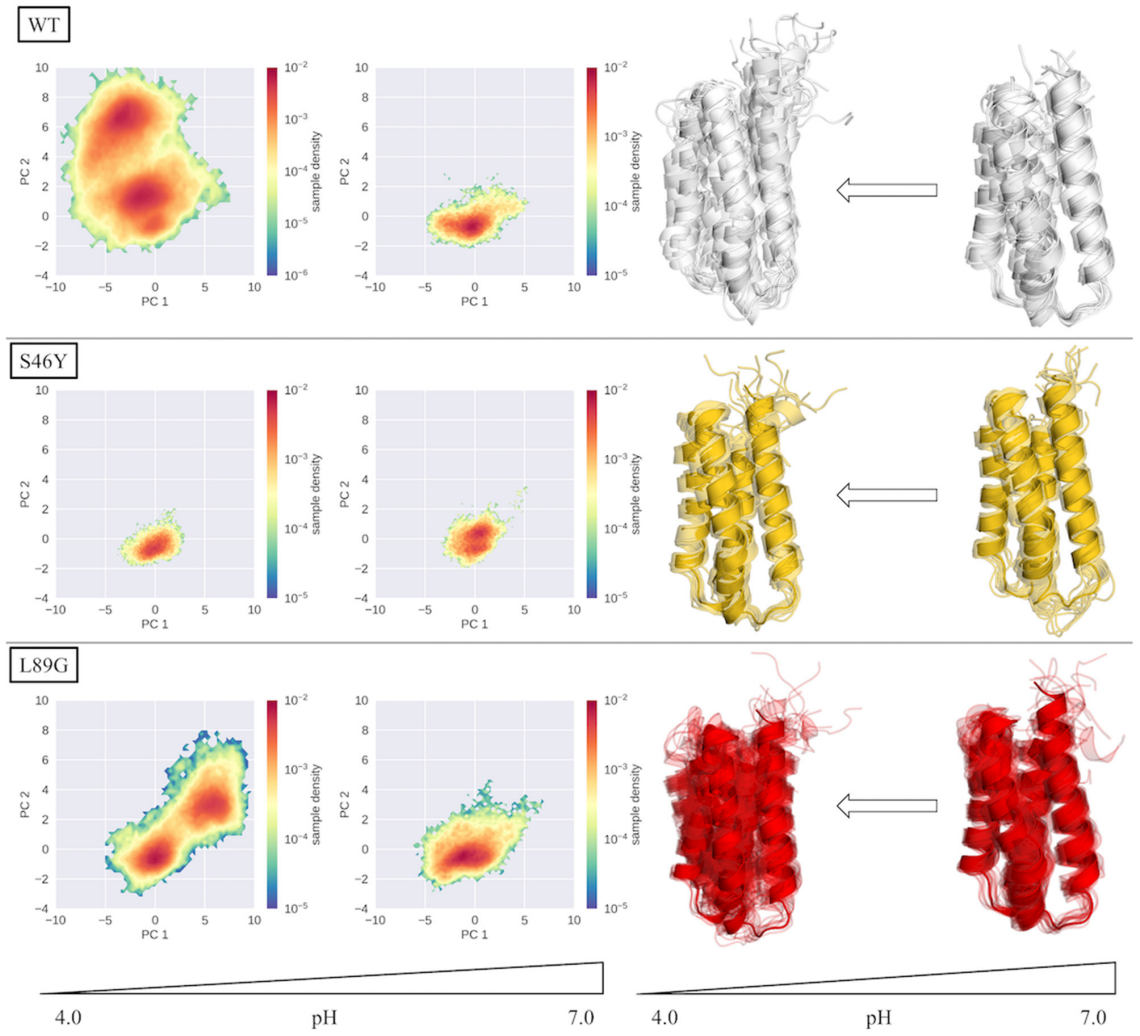
Overview of PCA spaces of the seeded simulations and the representatives of the ensembles of all simulated systems at pH 4.0 and 7.0. Structural ensembles of wild type and L89G mutant clearly become more diverse at lower pH values, while the S46Y mutant shows low structural flexibility at both pH values.

In summary, our results are perfectly in line with the observed experimental stabilities and proteolytic degradation patterns. Figure 6 recaps our findings, showing the PCA spaces of the seeded simulations on the left side and a representation of the conformational ensemble on the right side. At pH 7.0, the wild type and the S46Y mutant remain mostly stable and folded, while the L89G mutant is more flexible and shows local unfolding. At lower pH values the flexibilities and unfolding tendencies of the wild type and the L89G mutant increase significantly. In contrast to that, the thermally and proteolytically stabilized S46Y mutant shows only a slight increase in flexibility at lower pH values and mostly retains a stable fold. We surmise that local unfolding events captured in our models sufficiently open up the secondary structure elements of the protein to facilitate proteolytic cleavage. Both the location and the frequency of the unfolding events captured in our simulations are in line with the observed experimental cleavage patterns.

In conclusion, our approach provides valuable information in atomistic detail of the unfolding kinetics and differences therein of allergenic proteins. We are able to capture pH-induced local unfolding events, which can be directly linked to the experimentally observed cleavage patterns. Our workflow combining enhanced and classical MD simulations, with MSM analysis provides accurate thermodynamic and kinetic information of the underlying unfolding dynamics.

## Data Availability Statement

The original contributions presented in the study are included in the article/Supplementary Materials, further inquiries can be directed to the corresponding author/s.

## Author Contributions

All authors listed have made a substantial, direct and intellectual contribution to the work, and approved it for publication.

## Conflict of Interest

The authors declare that the research was conducted in the absence of any commercial or financial relationships that could be construed as a potential conflict of interest.
