# Supplementary material for: pH-Induced Local Unfolding of the Phl p 6 Pollen Allergen From cpH-MD
Source: Front Mol Biosci. 2021 Jan 12;7:603644. doi: 10.3389/fmolb.2020.603644 (PMC7835895; doi:10.3389/fmolb.2020.603644)
Supplement: Supplementary file 1 [file Table_1.DOCX]

***Supplementary Material***


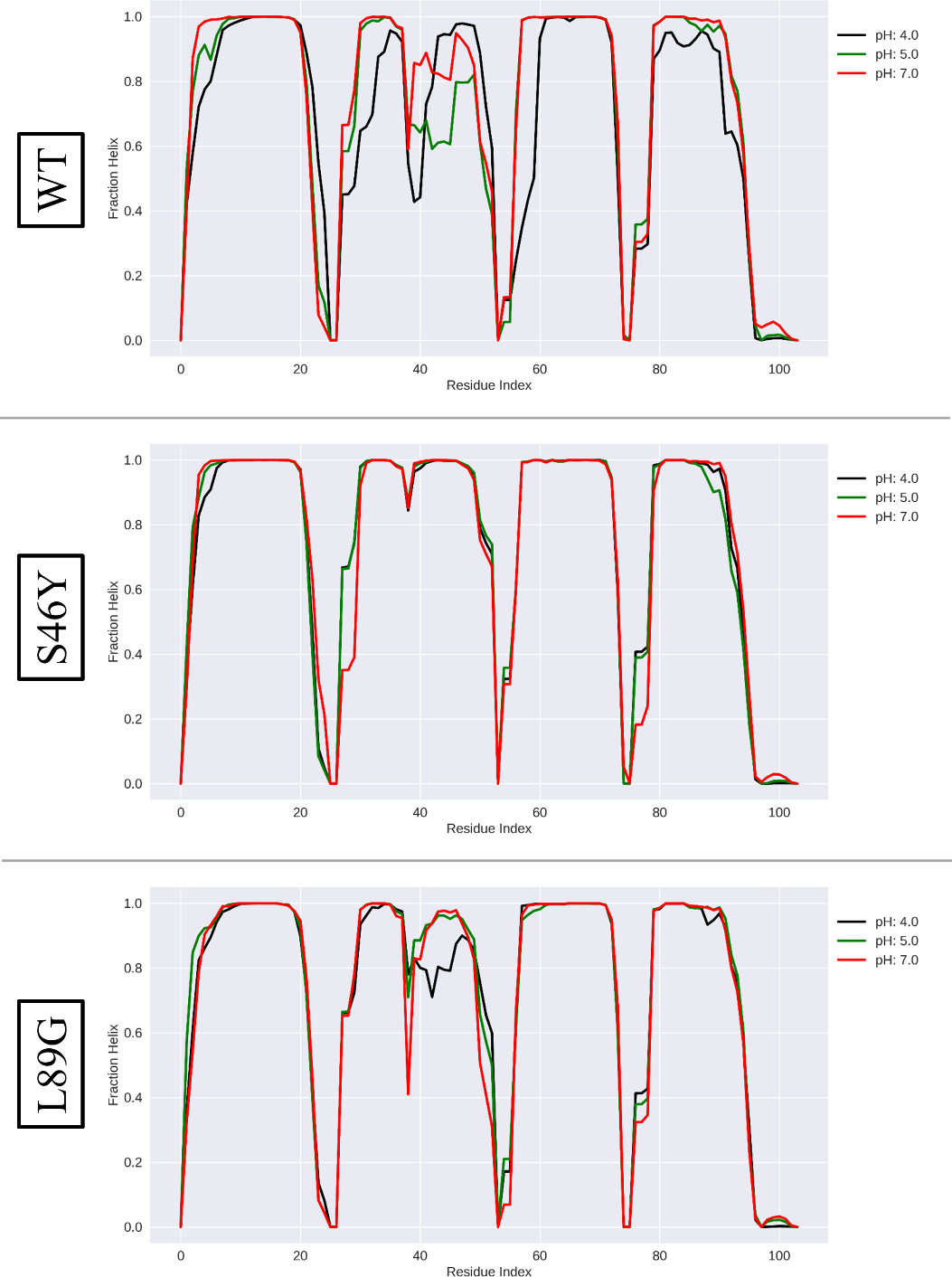


**Supplementary Figure 1**: Residue-wise fraction of helical content of the Phl p 6 wild type, S46Y and L89G mutant, captured in the cpH-aMD simulations.


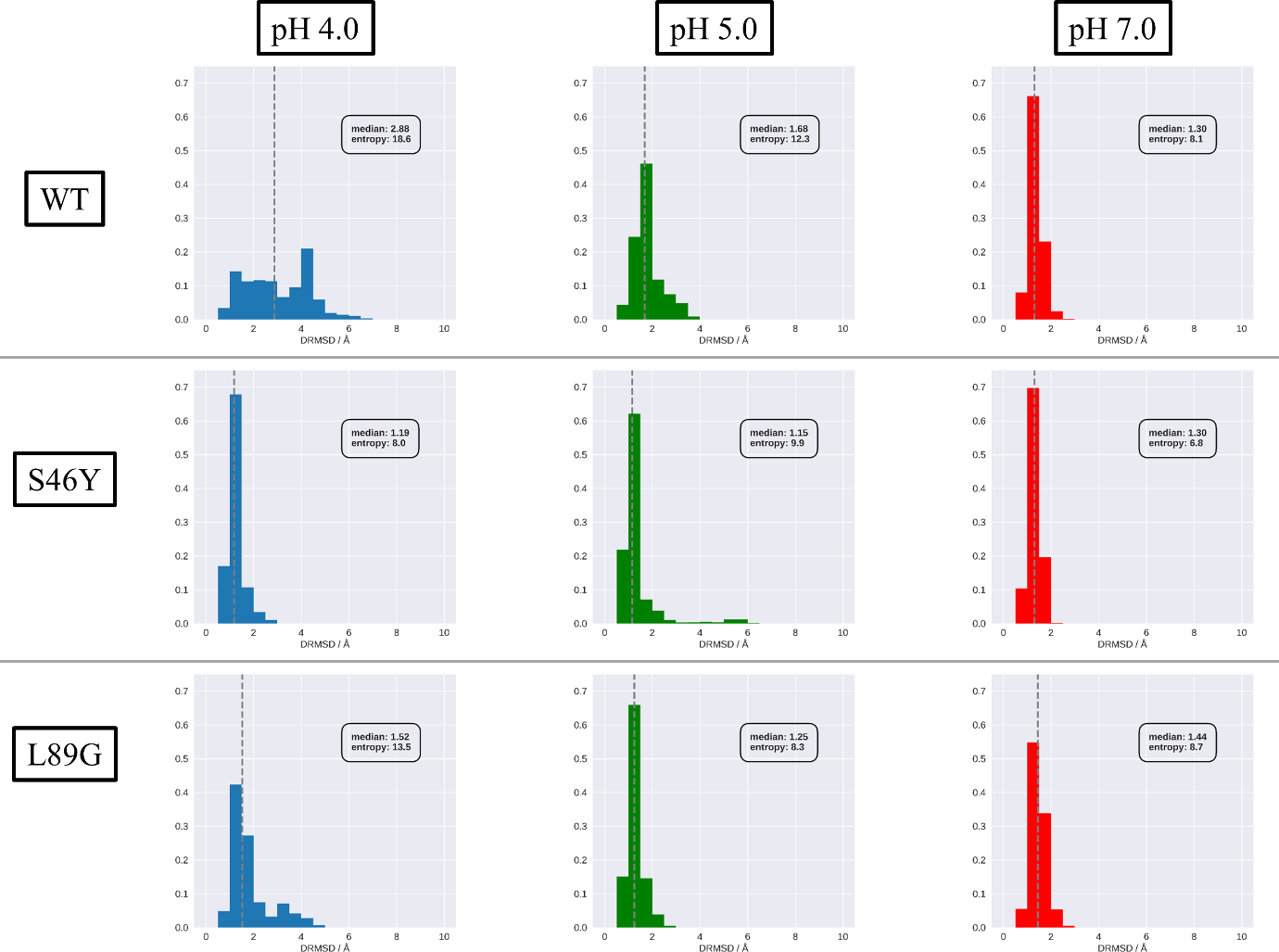


**Supplementary Figure 2**: DRMSD distributions captured in the cpH-aMD simulations for the Phl p 6 wild type, S46Y and L89G mutant at the simulated pH values.


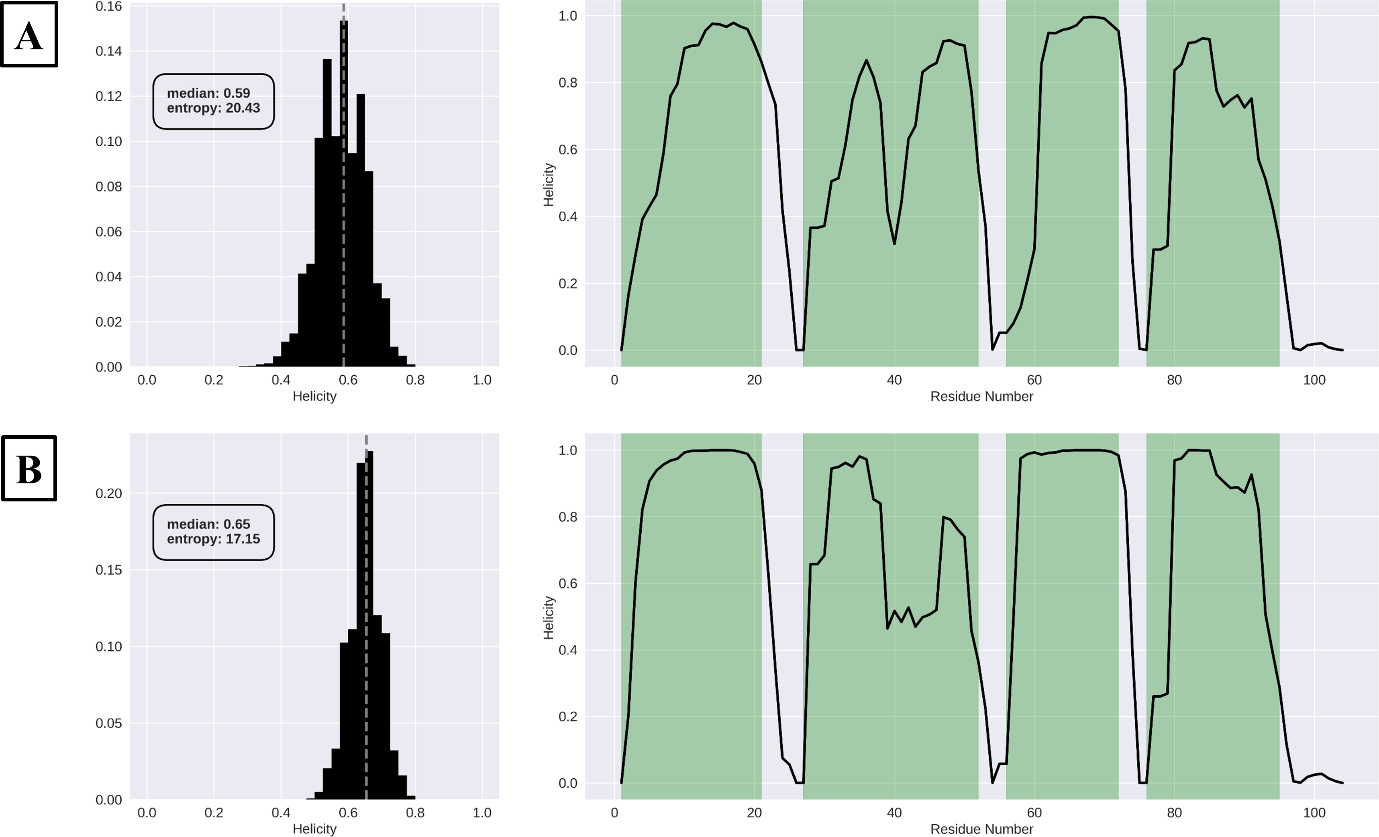


**Supplementary Figure 3**: Fraction of the helical content as overall histogram and per-residue plot from the seeded cpH-MD simulations of the Phl p 6 wild type at pH 4.0 (A) and pH 7.0 (B).


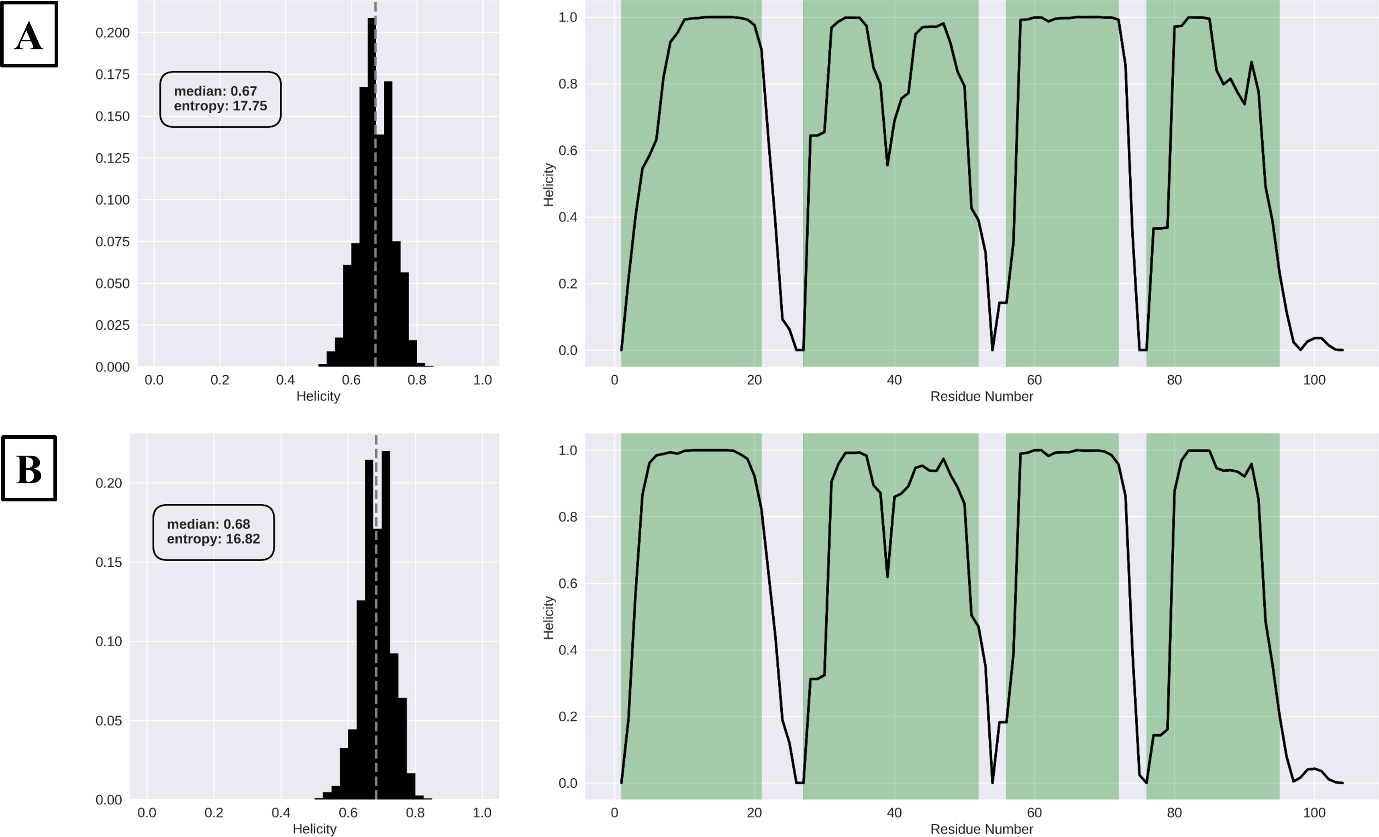


**Supplementary Figure 4**: Fraction of the helical content as overall histogram and per-residue plot from the seeded cpH-MD simulations of the Phl p 6 S46Y mutant at pH 4.0 (A) and pH 7.0 (B).


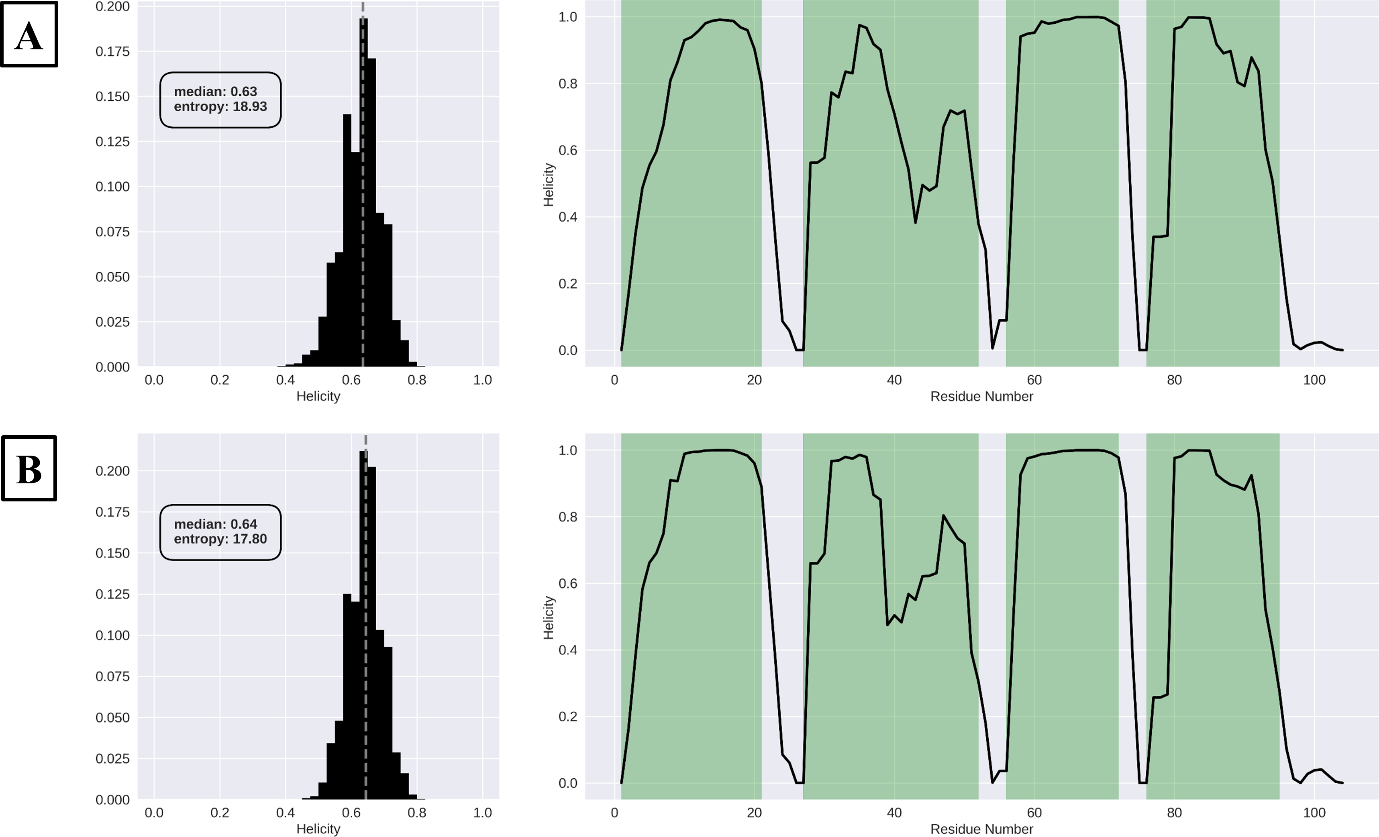


**Supplementary Figure 5**: Fraction of the helical content as overall histogram (A) and per-residue plot (B) from the seeded cpH-MD simulations of the Phl p 6 L89G mutant at pH 4.0 (A) and pH 7.0 (B).


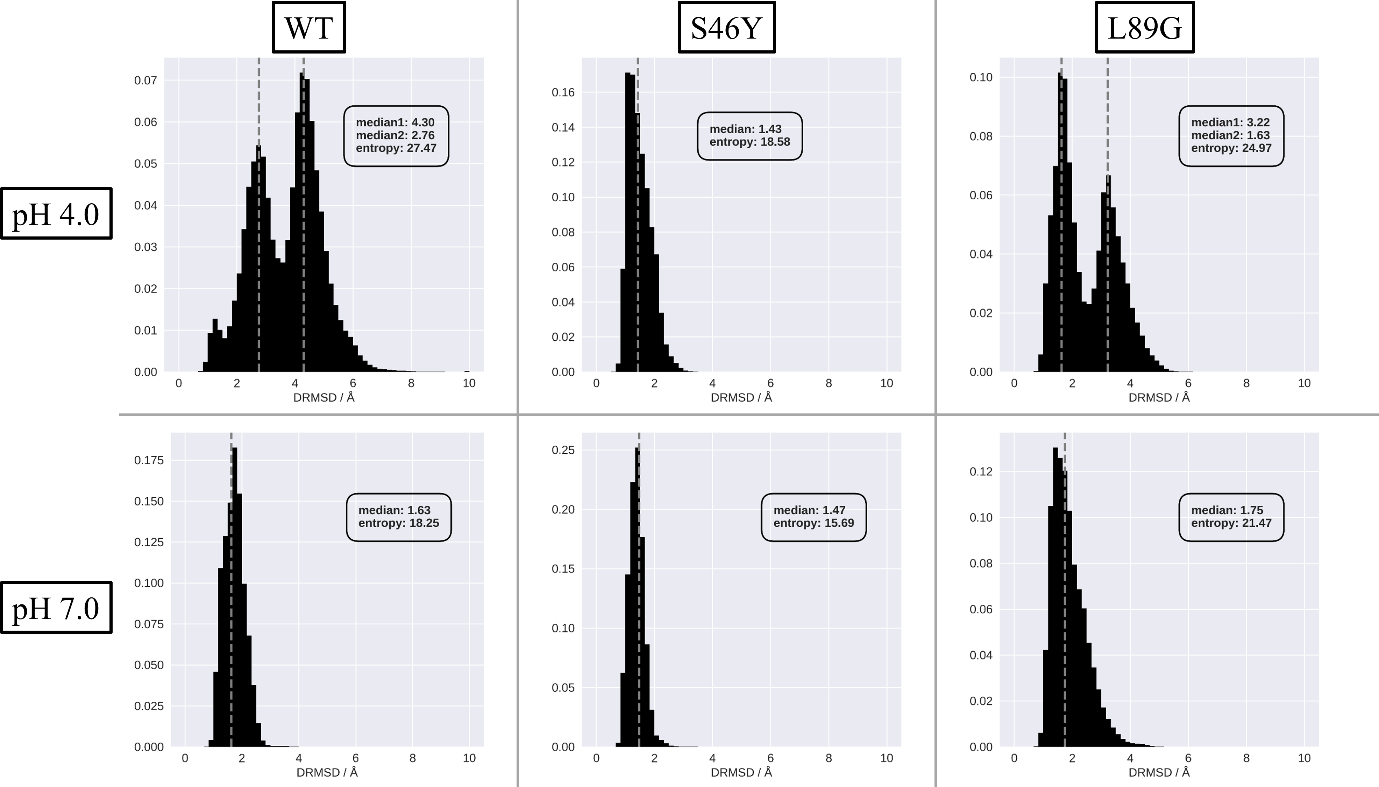


**Supplementary Figure 6**: DRMSD distributions at pH 4.0 and pH 7.0 of the seeded cpH-MD simulations. Heavy backbone atoms of residues 1-98 were considered for the calculation.


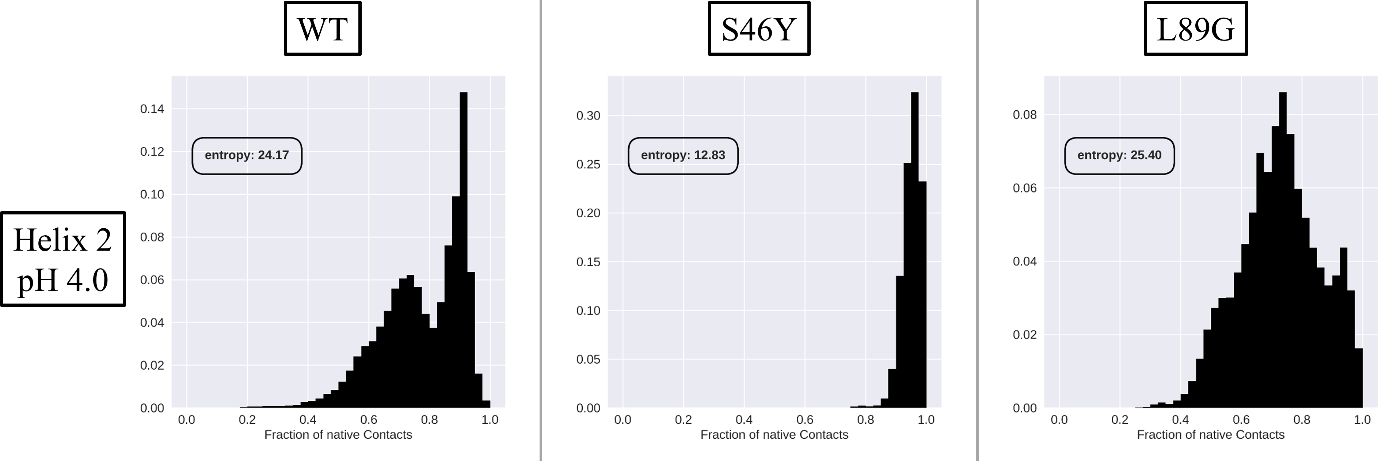


**Supplementary Figure 7**: Distributions of the fraction of native contacts of the second helix (residues 28-53) at pH 4.0 for the wild type, S46Y and L89G mutant. All heavy atom contacts were considered for the calculation.


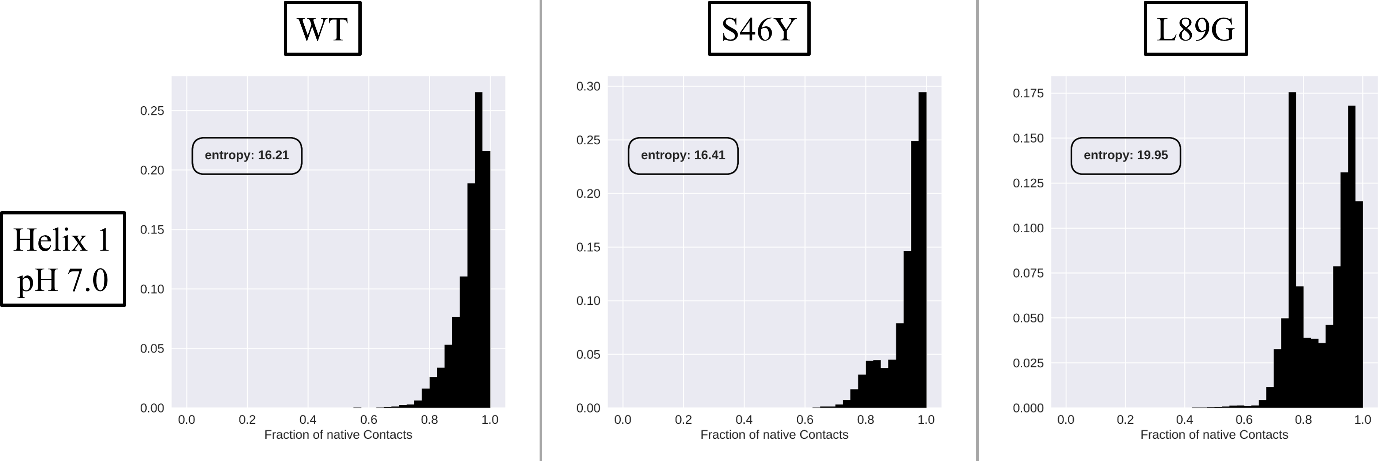


**Supplementary Figure 8**: Distributions of the fraction of native contacts of the first helix (residues 2-25) at pH 7.0 for the wild type, S46Y and L89G mutant. All heavy atom contacts were used for the calculation.


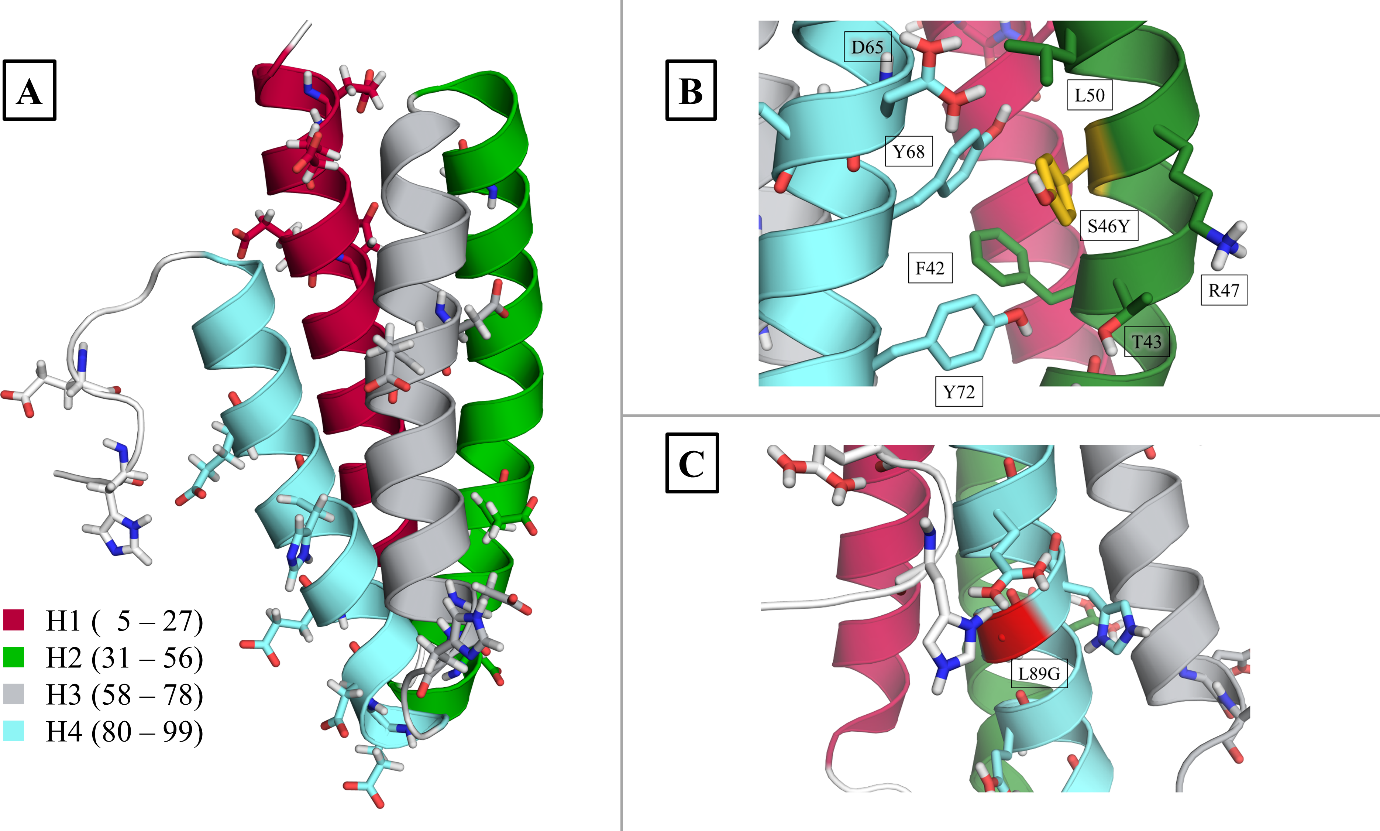


**Supplementary Figure 9**: Structural visualization of the Phl p 6 allergen. Wild type crystal structure (PDB 1NLX, chain A) with color-coded helices 1 to 4 and all titrated residues is shown in section A. Location of the S46Y and L89G mutation with all surrounding titrated residues are shown in sections B and C, respectively.

**Supplementary Table 1**: Macrostate populations of the Phl p 6 wild type, S46Y and L89G mutants.

| **Wild Type** | | | | | |
| --- | --- | --- | --- | --- | --- |
| pH | State 1 | State 2 | State 3 | State 4 | State 5 |
| 4.0 | 0.173 | 0.363 | 0.464 |  |  |
| 7.0 | 0.027 | 0.042 | 0.454 | 0.280 | 0.198 |
|  |  |  |  |  |  |
| **S46Y Mutant** | | | | | |
| pH | State 1 | State 2 | State 3 | State 4 |  |
| 4.0 | 0.008 | 0.653 | 0.126 | 0.214 |  |
| 7.0 | 0.018 | 0.178 | 0.804 |  |  |
|  |  |  |  |  |  |
| **L89G Mutant** | | | | | |
| pH | State 1 | State 2 | State 3 | State 4 |  |
| 4.0 | 0.026 | 0.352 | 0.385 | 0.237 |  |
| 7.0 | 0.037 | 0.065 | 0.422 | 0.476 |  |

**Supplementary Table 2**: Transition timescales between the MSM macrostates for the Phl p 6 wild type at pH 4.0.

| **WT pH 4.0** | | | |
| --- | --- | --- | --- |
| States | 1 | 2 | 3 |
| 1 |  | 177.94 | 288.94 |
| 2 | 959.6 |  | 63.71 |
| 3 | 1085.01 | 69.74 |  |

**Supplementary Table 3**: Transition timescales between the MSM macrostates for the Phl p 6 wild type at pH 7.0.

| **WT pH 7.0** | | | | | |
| --- | --- | --- | --- | --- | --- |
| States | 1 | 2 | 3 | 4 | 5 |
| 1 |  | 442.24 | 16.12 | 69.05 | 129.8 |
| 2 | 392.24 |  | 37.5 | 84.08 | 144.83 |
| 3 | 487.71 | 556.98 |  | 29.48 | 90.42 |
| 4 | 554.1 | 614.42 | 31.42 |  | 46.94 |
| 5 | 570.86 | 631.18 | 48.35 | 12.78 |  |

**Supplementary Table 4**: Transition timescales between the MSM macrostates for the S46Y mutant at pH 4.0.

| **S46Y pH 4.0** | | | | |
| --- | --- | --- | --- | --- |
| States | 1 | 2 | 3 | 4 |
| 1 |  | 8.91 | 149.21 | 24.47 |
| 2 | 385.95 |  | 207.83 | 8.5 |
| 3 | 373.26 | 37.61 |  | 45.59 |
| 4 | 398.04 | 2.93 | 211.28 |  |

**Supplementary Table 5**: Transition timescales between the MSM macrostates for the S46Y mutant at pH 7.0.

| **S46Y pH 7.0** | | | |
| --- | --- | --- | --- |
| States | 1 | 2 | 3 |
| 1 |  | 71.13 | 81.95 |
| 2 | 4176.53 |  | 36.29 |
| 3 | 4334.22 | 208.12 |  |

**Supplementary Table 6**: Transition timescales between the MSM macrostates for the L89G mutant at pH 4.0.

| **L89G pH 4.0** | | | | |
| --- | --- | --- | --- | --- |
| States | 1 | 2 | 3 | 4 |
| 1 |  | 29.84 | 6.11 | 73.4 |
| 2 | 283.89 |  | 17.87 | 84.75 |
| 3 | 293.85 | 50.13 |  | 30.95 |
| 4 | 312.37 | 66.24 | 7.02 |  |

**Supplementary Table 7**: Transition timescales between the MSM macrostates for the L89G mutant at pH 7.0.

| **L89G pH 7.0** | | | | |
| --- | --- | --- | --- | --- |
| States | 1 | 2 | 3 | 4 |
| 1 |  | 69.45 | 8.75 | 38.91 |
| 2 | 125.52 |  | 6.31 | 41.04 |
| 3 | 127.35 | 69.72 |  | 30.26 |
| 4 | 160.18 | 107.22 | 30.33 |  |

**Supplementary Table 8**: Average protonation of all titrated residues of the seeded simulations at both simulated pH values.

|  | **pH 4.0** | | |  |  | **pH 7.0** | | |
| --- | --- | --- | --- | --- | --- | --- | --- | --- |
| **Residue** | **WT** | **S46Y** | **L89G** |  | **Residue** | **WT** | **S46Y** | **L89G** |
| GLU7 | 0.65 | 0.60 | 0.61 |  | GLU7 | 0.01 | 0.01 | 0.01 |
| GLU8 | 0.75 | 0.61 | 0.68 |  | GLU8 | 0.02 | 0.01 | 0.02 |
| GLU13 | 0.66 | 0.69 | 0.66 |  | GLU13 | 0.03 | 0.04 | 0.03 |
| ASP14 | 0.35 | 0.33 | 0.38 |  | ASP14 | 0.00 | 0.00 | 0.00 |
| ASP33 | 0.53 | 0.80 | 0.70 |  | ASP33 | 0.02 | 0.02 | 0.02 |
| GLU39 | 0.89 | 0.99 | 0.95 |  | GLU39 | 0.06 | 0.06 | 0.06 |
| ASP52 | 0.11 | 0.21 | 0.15 |  | ASP52 | 0.00 | 0.00 | 0.00 |
| ASP65 | 0.25 | 0.38 | 0.32 |  | ASP65 | 0.00 | 0.00 | 0.00 |
| GLU66 | 0.74 | 0.74 | 0.76 |  | GLU66 | 0.02 | 0.02 | 0.02 |
| ASP76 | 0.31 | 0.33 | 0.35 |  | ASP76 | 0.00 | 0.00 | 0.00 |
| HIS77 | 1.00 | 1.00 | 1.00 |  | HIS77 | 0.68 | 0.67 | 0.68 |
| GLU81 | 0.84 | 0.72 | 0.78 |  | GLU81 | 0.01 | 0.09 | 0.01 |
| ASP82 | 0.64 | 0.84 | 0.83 |  | ASP82 | 0.16 | 0.16 | 0.12 |
| GLU85 | 0.60 | 0.50 | 0.59 |  | GLU85 | 0.09 | 0.07 | 0.10 |
| HIS90 | 0.99 | 0.99 | 0.99 |  | HIS90 | 0.24 | 0.23 | 0.28 |
| GLU93 | 0.57 | 0.62 | 0.63 |  | GLU93 | 0.02 | 0.01 | 0.01 |
| GLU103 | 0.78 | 0.77 | 0.76 |  | GLU103 | 0.03 | 0.03 | 0.02 |
| HIS105 | 0.99 | 0.98 | 0.99 |  | HIS105 | 0.35 | 0.36 | 0.37 |
